# Opacities of the Cornea

**Published:** 1891-12-26

**Authors:** 


### OPHTHALMOLOGY.

### OPACITIES OF THE CORNEA.

Opacities of the cornea arise at the seat of ulcers, and are really the cicatrices of healed ulcers, whether from traumatic or constitutional causes, or are due to inflammations of the cornea, local or diffuse Keratitis, or actual deposits, such as lead salts (the carbonate of lead), precipitated from eye washes on to the abraded cornea, or from primary calcareous films. The opacity varies in depth, according to the depth of corneal tissue affected, and in its interference with vision according to the position of the opacity, which, if on the centre of the cornea, opposite the pupillary aperture, must obstruct the rays of light passing to the macula lutea, whereas, if situated near the margin of the cornea it will only obstruct those rays passing to the peripheral parts of the retina, which are not so important for clear vision.

When the superficial part of the cornea is rendered cloudy, the cloudiness is called a *Nebula*, only the epithelium and Bowman's membrane being affected. It is often too faint to be discovered by the unaided eye; a patient may come complaining of the dimness of vision in one eye, and on using Snellen's types is found to see better with the other eye; glasses spherical or cylindrical may improve the sight, but not bring it up to the normal standard. If the patient be now examined with a bright oblique illumination by focussing with a lens, the light from a lamp placed in front, and somewhat to the side of the patient, on to the cornea, a cloudiness, as if the cornea had been breathed upon, will be discovered opposite the centre of the pupil, and thus accounting for the diminution of vision. If not centrally situated, a nebula will probably not be a source of any inconvenience, and its existence may pass undiscovered until the eye be examined for some other cause. When the opacity is clearly visible to the naked eye, and of a milky white appearance it is known as *Leucoma*; if centrally situated it will be a source of great obstruction to sight, rendering the eye practically useless, as regards its central vision, if on the periphery of the cornea, though not obstructing central vision, it will cut off some rays of light, and also demands attention as being a source of much disfigurement.

The opacities from Keratitis, the result of constitutional dyscrasiæ, require the treatment specific to the disease, and no surgical interference should be allowed until all signs of further absorption of the opacity have ceased, when it may be classed with a chronic leucoma. *Nebulæ*, when slight, will often disappear by the use of massage combined with weak mercurial ointment, or calomel dusted into the eye, massage being applied at once. Cineraria maritima, a drug recently much vaunted as a cure for cataract, has been certainly useful in these cases, and as the laity often call opacities of the cornea, *cataracts*, this may account for its reputation in curing them. *Leucomata* are not likely to clear altogether, though the vision may improve considerably under the above treatment. It is, therefore, necessary to consider what can be done surgically, first to improve the vision, second to improve the appearance of the eye.

To improve vision, an artificial pupil may be made by removing a portion of the iris, as in the operation of Iridectomy. As a preliminary step the pupil should be dilated fully by atropine, after which the cornea must be carefully inspected by aid of the oblique light to ascertain which is its most transparent portion, and the eye tested for distant vision; if the latter be improved the operation should be decided on. If one has the choice of sites for the operation, the new pupil should be made downwards and inwards, or downwards, and only sufficient of the iris to make a useful pupil should be removed.

The eye being anæsthetised by cocain, and the lids separated by a spring speculum, the eyeball is grasped by fixation forceps, which the surgeon holds in one hand while he makes his incision with a keratome held in the other hand, through the corneal margin into the anterior chamber, the incision being about three mm. in extent; he hands the forceps to an assistant, and seizes the free margin of the iris with iris forceps, or a Tyrrel's hook, and drags the portion to be removed through the corneal wound, and snips it off with scissors at each angle of the wound; the instruments are removed, atropine instilled, the lids closed, and a pad and bandage applied.

If the leucoma admit enough rays of light to make vision through the artificial pupil confusing, the opacity may be tatooed; this is also the cosmetic treatment of leucomata. The operation may be done at one sitting, but as it is painful the eye should be well cocainised, the eyelids held open by a speculum, and the globe fixed with toothed forceps held by the surgeon with one hand; with the other he makes, either by means of a sharp needle or the special tatooing instrument, a large number of scratches over the opacity, and then rubs in a preparation of Indian ink finely powdered and made into a thick paste with water.

Transplantation of the cornea, or the removal of the opaque portion, and substitution of a clear piece of cornea from a rabbit has been successfully performed. The operation was originated by Dr. I. R. Wolfe, of Glasgow, who devotes a chapter to it in his book on "Diseases and Injuries of the Eye," and which will repay reading by anyone interested in the operation. He has also used a healthy piece of human cornea from an eye that had to be removed, and successfully transplanted it on to another human cornea.

In the case of lead deposits the part must be carefully scraped at repeated sittings by means of a small sharp scoop, or the part where the deposit is, removed *en masse* by a Beer's knife, the eye dressed afterwards with cocain dissolved in castor oil, and a pad applied to the closed lids.

The calcareous deposits are treated in the same manner, and have been found to consist of calcium phosphate and calcium carbonate (Nettleship). It occurs in persons afflicted with gouty dyscrasia, but has not yet been proved to be in direct connection with it (Hutchinson). The cornea has also been stained by the use of strong solutions of nitrate of silver. After a single application of strong nitrate of silver solution, the cornea is often rendered cloudy immediately, a milky-white opacity which, however, disappears in the course of a few days. A solution of chloride of sodium should always be used immediately after such application.

